# ATP-dependent polymerization dynamics of bacterial actin proteins involved in *Spiroplasma* swimming

**DOI:** 10.1098/rsob.220083

**Published:** 2022-10-26

**Authors:** Daichi Takahashi, Ikuko Fujiwara, Yuya Sasajima, Akihiro Narita, Katsumi Imada, Makoto Miyata

**Affiliations:** ^1^ Graduate School of Science, Osaka Metropolitan University, Osaka, Japan; ^2^ The OMU Advanced Research Center for Natural Science and Technology, Osaka Metropolitan University, Osaka, Japan; ^3^ Graduate School of Science, Osaka City University, Osaka, Japan; ^4^ The OCU Advanced Research Institute for Natural Science and Technology (OCARINA), Osaka City University, Osaka, Japan; ^5^ Department of Materials Science and Bioengineering, Nagaoka University of Technology, Nagaoka, Niigata, Japan; ^6^ Graduate School of Science, Nagoya University, Nagoya, Japan; ^7^ Graduate School of Science, Osaka University, Toyonaka, Japan

**Keywords:** MreB, bacterial cytoskeleton, X-ray crystallography, electron microscopy, Mollicutes, cell motility

## Abstract

MreB is a bacterial protein belonging to the actin superfamily. This protein polymerizes into an antiparallel double-stranded filament that determines cell shape by maintaining cell wall synthesis. *Spiroplasma eriocheiris*, a helical wall-less bacterium, has five MreB homologous (SpeMreB1-5) that probably contribute to swimming motility. Here, we investigated the structure, ATPase activity and polymerization dynamics of SpeMreB3 and SpeMreB5. SpeMreB3 polymerized into a double-stranded filament with possible antiparallel polarity, while SpeMreB5 formed sheets which contained the antiparallel filament, upon nucleotide binding. SpeMreB3 showed slow P_i_ release owing to the lack of an amino acid motif conserved in the catalytic centre of MreB family proteins. Our SpeMreB3 crystal structures and analyses of SpeMreB3 and SpeMreB5 variants showed that the amino acid motif probably plays a role in eliminating a nucleophilic water proton during ATP hydrolysis. Sedimentation assays suggest that SpeMreB3 has a lower polymerization activity than SpeMreB5, though their polymerization dynamics are qualitatively similar to those of other actin superfamily proteins, in which pre-ATP hydrolysis and post-P_i_ release states are unfavourable for them to remain as filaments.

## Graphical abstract

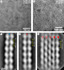

## 1. Introduction

MreB is an actin superfamily protein that is found in most elongated bacteria [1,2]. This protein polymerizes into an antiparallel double-stranded filament, and the polymerization depends on the binding of nucleotides, such as ATP and GTP [3–7]. The conformational change of MreB upon polymerization induces the rearrangement of the conserved glutamate and threonine residues interacting with the putative nucleophilic water for γ-P_i_ of the bound nucleotide, thereby facilitating nucleotide hydrolysis [4,8]. Although the residues important for nucleotide hydrolysis are conserved in many MreBs [4,9], the hydrolysis mechanism and the role of hydrolysis in polymerization dynamics remain unclear.

In walled bacteria, MreB functions as a scaffold for an ‘elongasome' complex involved in the synthesis of the peptidoglycan layer, the bacterial cell wall, during the cell elongation phase. MreB causes the bacterial cells to be arranged in a rod shape [2,10]. MreB forms filaments (with very slow subunit turnover) on the cell membrane. These filaments move in a direction perpendicular to the cell axis. Importantly, filament movement is coupled with peptidoglycan synthesis, rather than polymerization dynamics [11,12]. However, some MreBs play roles distinct from cell wall synthesis, for example, *Myxococcus xanthus* MreB drives cell gliding [13], and *Helicobacter pylori* MreB is involved in chromosome segregation and urease activity [14]. Recently, MreB proteins involved in swimming motility were identified in *Spiroplasma* species [9,15].

*Spiroplasma* belongs to the class Mollicutes, which evolved from the phylum Firmicutes, which includes *Bacillus subtilis* [16–18]. *Spiroplasma* has helical-shaped cells lacking the peptidoglycan layer and show unique swimming motility, in which the cell moves forward by transmitting helicity switching along the cell axis to rotate the cell body. This motility is unrelated to major bacterial motilities such as flagellar and pili motilities [16,19–22]. Helicity switching and its transmission are likely caused by conformational changes in the internal helical ribbon structure along the entire cell axis [19,23–25]. The ribbon structure is thought to be composed of fibril, a cytoskeletal protein unique to *Spiroplasma* [19,23,24], and five classes of MreB proteins (MreB1-5) [25–27]. A recent structural study on *Spiroplasma* *citri* MreB5 (SciMreB5) showed that SciMreB5 has a canonical actin fold and forms filaments [3].

Based on sequence similarity, the five MreBs were divided into three functional groups: MreB1&4, MreB2&5 and MreB3 (electronic supplementary material, figure S1A) [26]. A recent study proposed functions for each MreB group using a heterologous expression system as follows. MreB1 and/or MreB4 form a static backbone that interacts with fibril filaments along the cell, MreB2 and/or MreB5 actively polymerize and depolymerize to change the ribbon conformation, and MreB3 anchors MreB1 and/or MreB4 onto the cell membrane via its amphipathic helix (electronic supplementary material, figure S1B) [26]. MreB5 is essential for the helical cell shape and swimming motility of *S. citri* [3]. To understand the swimming mechanism, it is necessary to clarify the molecular features of MreBs, including their structure, ATPase activity and polymerization dynamics. Here, we studied MreB3 and MreB5 in *Spiroplasma eriocheiris* (SpeMreB3 and SpeMreB5, respectively), a model organism for studying *Spiroplasma* swimming [19,20,23,28,29]. SpeMreB3 polymerizes into a double-stranded filament with possible antiparallel polarity, while SpeMreB5 forms a sheet which contains the antiparallel filament upon nucleotide binding. Our SpeMreB3 crystal structures of SpeMreB3 and P_i_ release measurements suggest that SpeMreB3 lacks an amino acid motif for ATP hydrolysis that is conserved in other MreB family proteins, resulting in low ATPase activity. These differences between SpeMreB3 and SpeMreB5 are likely essential for their distinct cellular functions. Our data also suggest the following two molecular features of MreBs: (1) an ATP hydrolysis mechanism in MreB family proteins, including a possible proton transfer pathway and (2) the polymerization dynamics of SpeMreB3 and SpeMreB5, which are qualitatively similar to other actin superfamily proteins [30].

## 2. Results

### 2.1. Nucleotide binding induces SpeMreB3 and SpeMreB5 polymerization into double-stranded filaments and asymmetric sheets, respectively

**Figure 1.**
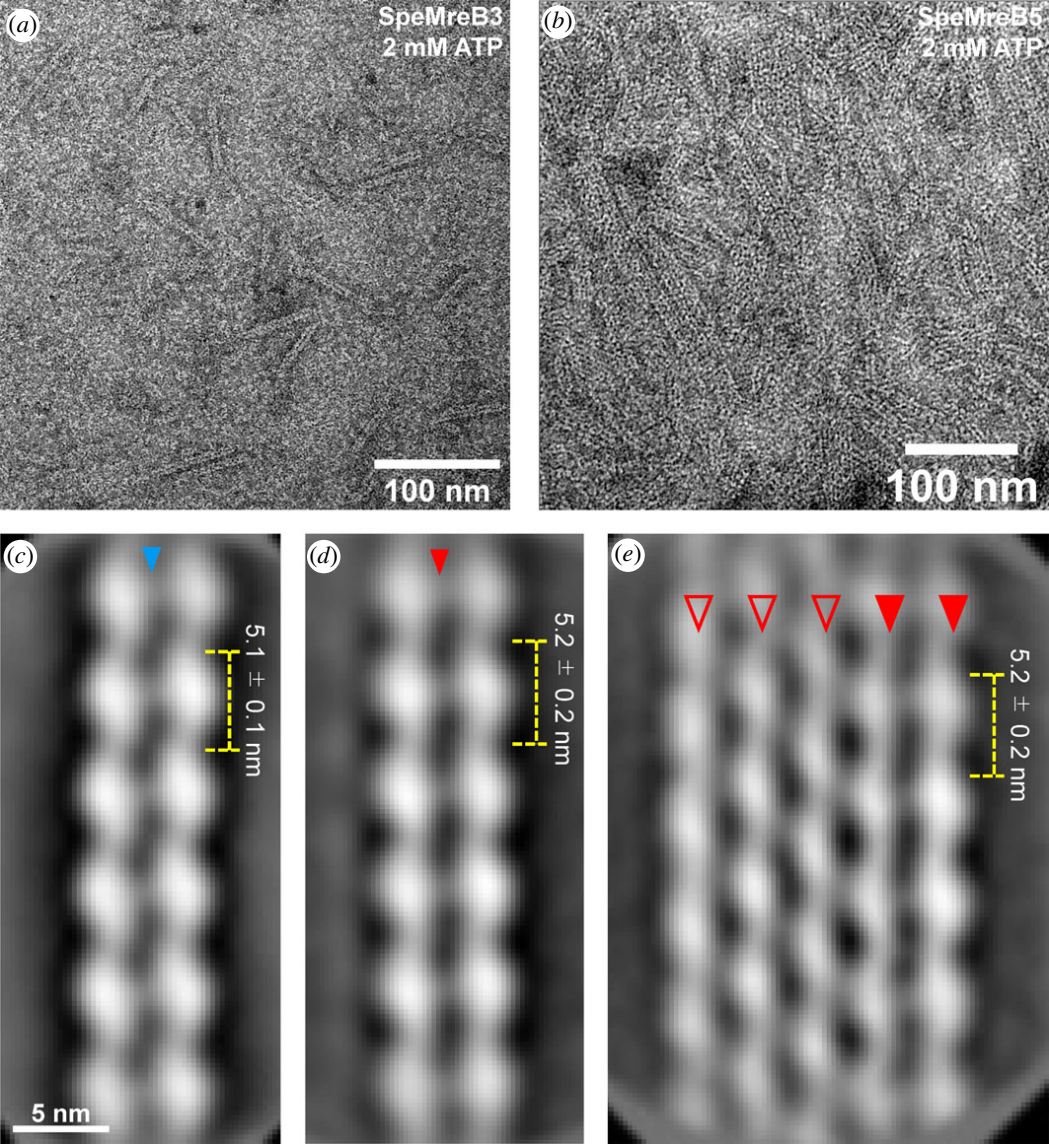
Structures of SpeMreB3 and SpeMreB5 filaments observed by EM. (*a,b*) Negative-staining EM image of (*a*) 10 µM SpeMreB3 and (*b*) 5 µM SpeMreB5. The samples were diluted to 3 µM prior to placement onto an EM grid. (*c,d*) Two-dimensional averaged image of (*d*) SpeMreB3 and (*d*) SpeMreB5 filaments averaged from 2874 to 652 particles, respectively. The estimated subunit repeats are 5.1 ± 0.1 and 5.2 ± 0.2 nm for SpeMreB3 and SpeMreB5, respectively. A weak electron density connecting the protofilaments is indicated by a triangle. (*e*) A two-dimensional averaged image of the five-stranded SpeMreB5 sheet structure averaged from 1575 particles. The estimated subunit repeat is 5.2 ± 0.2 nm. The protofilaments in the juxtaposed filament and the other protofilaments are indicated by solid and open triangles, respectively.

We individually expressed all five SpeMreBs in *Escherichia coli* as fusions with a 6 × His-tag. SpeMreB1, SpeMreB2 and SpeMreB4 were not obtained from the soluble fraction. By contrast, SpeMreB3 and SpeMreB5 were soluble and were successfully purified as monomers (electronic supplementary material, figure S2), enabling us to assay the polymerization reactions. These SpeMreBs were individually incubated with or without 2 mM Mg-ATP in standard buffer (20 mM Tris–HCl pH 7.5, 100 mM KCl and 5 mM DTT) and were imaged using negative-staining electron microscopy (EM). In the presence of Mg-ATP, SpeMreB3 polymerized into double-stranded filaments approximately 100 nm in length and without helicity (figure 1*a*). This structure is comparable to that of *Caulobacter crescentus* MreB (CcMreB) filaments [4]. By contrast, SpeMreB5 formed sheet structures composed of multiple protofilaments with sub-micrometre length and width (figure 1*b*). We did not find single protofilament structures for either SpeMreB, suggesting the necessity of inter-protofilament interactions in the assembly. Filamentous structures were not observed in the absence of Mg-ATP (electronic supplementary material, figure S3A-B), indicating that polymerization is nucleotide-dependent.

To elucidate the subunit arrangement in these structures, we averaged the EM images using RELION v. 3.1 or 4.0 software [31]. We selected 13 077 SpeMreB3 and 117 740 SpeMreB5 images and obtained SpeMreB3 and SpeMreB5 filaments (figure 1*c*,*d*) and SpeMreB5 sheets (figure 1*e*; electronic supplementary material, figure S4J). The SpeMreB3 and SpeMreB5 filaments showed subunit repeats of 5.1 ± 0.1 and 5.2 ± 0.2 nm, respectively (figure 1*c*). The two protofilaments were linked via a weak density in a juxtaposed manner. These features resemble those of CcMreB filaments [4]. The SpeMreB5 sheets consisted of variable numbers of protofilaments aligned in a staggered manner (figure 1*e*; electronic supplementary material, figure S4J, open triangles) with a juxtaposed protofilament pair on one side (figure 1*e*; electronic supplementary material, figure S4J, solid triangles). The image of juxtaposed protofilament pair tended to be sharper than the other strands, indicating that the interactions to form juxtaposed pairs are less flexible than those of the staggered pair. Neither sheets composed of staggered protofilament pairs nor sheet composed of juxtaposed protofilament pairs were observed. Moreover, none of the sheets had juxtaposed double-stranded filaments on both sides simultaneously. These results indicate that the SpeMreB5 sheets are highly asymmetric and are composed of two distinct sets of inter-protofilament interactions.

To assess the polarity of these structures, we processed the two-dimensional (2D) averaged images. We first fitted each subunit density into an ellipse to determine each subunit axis, showing that the angles of all subunit axes are common for the juxtaposed filaments and the SpeMreB5 sheet (electronic supplementary material, figure S4A, D and H). Next, to assess the protofilament structural anisotropy, we overlaid two corresponding juxtaposed protofilament pairs. One pair was unprocessed and the other was rotated 180° or translated along the *x*-axis. The images rotated by 180° fit well with the original images (electronic supplementary material, figure S4B, E and L), indicating that the juxtaposed pairs possess rotational symmetry and that the projection angle is only slightly different between the protofilaments in each pair. The SpeMreB3 filament exhibited mismatches at the pole regions (electronic supplementary material, figure S4B), reflecting filament bending. The juxtaposed protofilament pair in the SpeMreB5 sheet shows that the density of the edge-most protofilament is more evident than that of the adjacent protofilament, probably because of the flexibility of the adjacent protofilament (electronic supplementary material, figure S4I). However, the overlay of the translated SpeMreB5 images shows mismatches in the intra-protofilament interaction regions (electronic supplementary material, figure S4F and M), indicating that the protofilaments are asymmetric in the transverse direction. These results indicate that the juxtaposed protofilament pair of SpeMreB5 possesses antiparallel polarity, similar to the CcMreB filament (electronic supplementary material, figure S4G-I) [4]. Although the mismatches in the overlay of the translated SpeMreB3 image were less obvious than those of SpeMreB5 (electronic supplementary material, figure S4C, F and M), antiparallel polarity is the most plausible arrangement for the SpeMreB3 filament, as it resembles the antiparallel filaments of SpeMreB5 and CcMreB. We also overlaid two SpeMreB5 sheet images, one of which was unprocessed and the other was translated along the *x*- and *y*-axes. The protofilament adjacent to the antiparallel protofilament pair fit well with the neighbouring protofilaments on both sides (electronic supplementary material, figure S4N), indicating that the staggered protofilaments were aligned in a parallel manner (electronic supplementary material, figure S4I).

Filament formation by *E. coli* MreB (EcMreB) requires nucleotide hydrolysis [5]. To determine whether this was also the case for SpeMreB3 and SpeMreB5, we conducted negative-staining EM of SpeMreB3 and SpeMreB5 incubated in the presence of 2 mM Mg-AMPPNP or Mg-ADP. SpeMreB3 incubated with Mg-AMPPNP or Mg-ADP formed double-stranded filaments (electronic supplementary material, figure S3C and D), while SpeMreB5 incubated with Mg-AMPPNP or Mg-ADP formed sheet structures (electronic supplementary material, figure S3E and F). These results indicate that SpeMreB3 and SpeMreB5 polymerization is driven by nucleotide binding, rather than hydrolysis.

### 2.2. SpeMreB3 crystal structure

**Figure 2.**
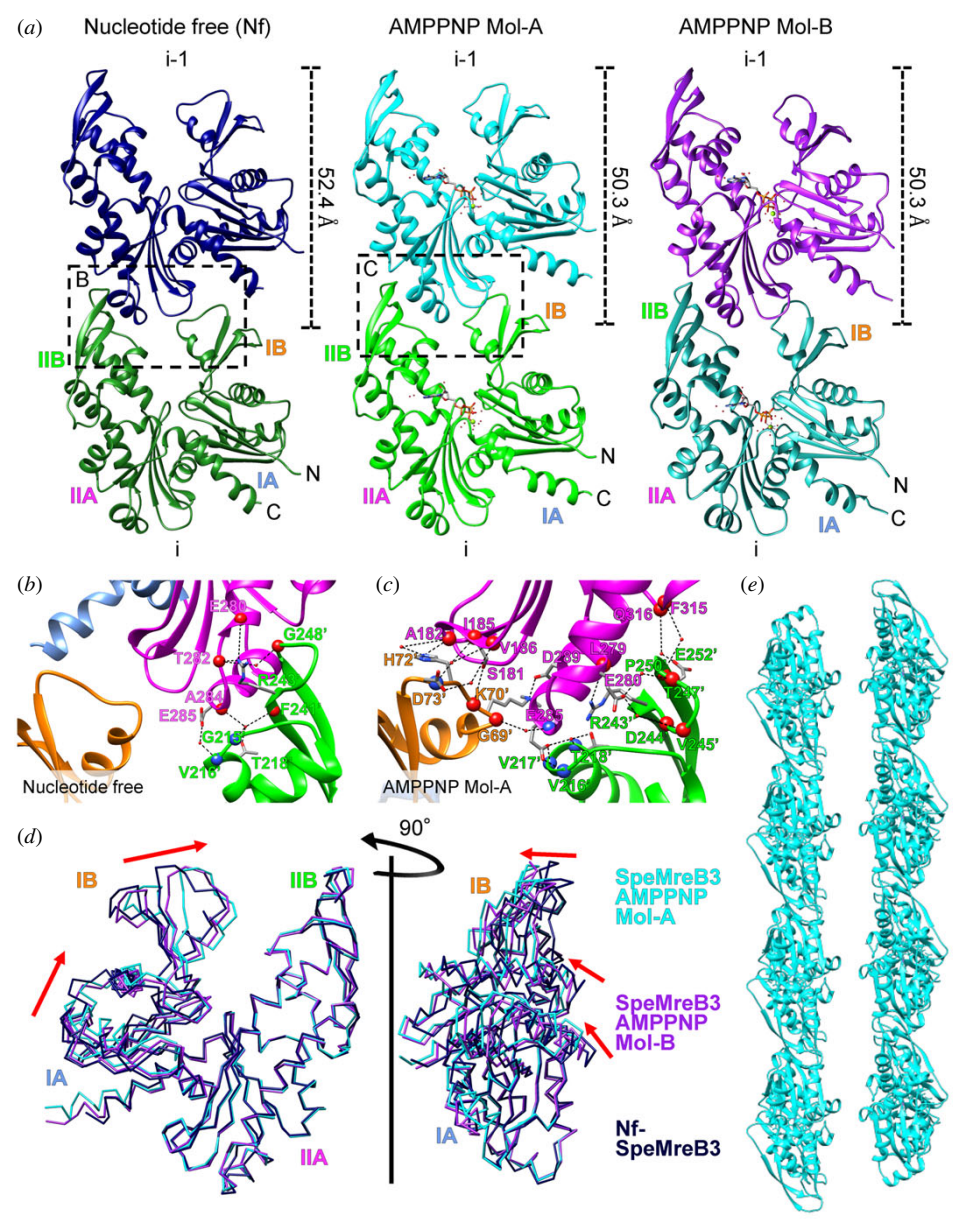
Crystal structures of SpeMreB3. (*a*) Protofilament structures in crystals of Nf-SpeMreB3 and SpeMreB3 AMPPNP complexes. Two different conformations (Mol-A and B) in the asymmetric unit of the SpeMreB3 AMPPNP complex crystal are shown in the centre and right panels, respectively. Two subunits in the protofilaments are labelled as i and i-1. The subunit repeat is indicated at the right of each i-1 subunit. The four subdomains (IA, IB, IIA and IIB) and N- and C-termini are labelled on the i subunit. The boxed regions on Nf and AMPPNP complex Mol-A protofilaments are magnified in *b* and *c*, respectively, to represent the intra-protofilament interactions. (*b,c*) Close up view of the subunit interface in protofilaments in the crystal of (*b*) Nf-SpeMreB3 and (*c*) the SpeMreB3 AMPPNP complex Mol-A. Subdomains IA, IB, IIA and IIB are indicated by ribbon representations coloured with light blue, orange, magenta and green, respectively. Hydrogen bonds and electrostatic interactions are indicated by broken lines. The residues involved in the hydrogen bonding or electrostatic interaction network are indicated by stick models or blue (backbone nitrogen atom) or red (backbone oxygen atom) spheres with labels. Water molecules involved in the interactions are shown as small red spheres. (*d*) Structural comparison of Mol-A and B in the SpeMreB3 AMPPNP complex and Nf-SpeMreB3. The structures are superimposed onto the subdomains IIA and IIB of Mol-A. The movement of the subdomain IA and IB relative to Nf-SpeMreB3 is indicated by red arrows. (*e*) A ribbon representation of the filament structure of SpeMreB3 AMPPNP Mol-A fit to the EM image (figure 1*c*; electronic supplementary material, figure S6E).

SpeMreB3 crystals suitable for X-ray experiments grew under several conditions but they showed merohedral twinning. To overcome this problem, we methylated the lysine residues of SpeMreB3 and crystallized the modified protein (electronic supplementary material, figure S5A and B) [32,33]. The crystal structures of nucleotide-free (Nf) SpeMreB3 and its AMPPNP complex were determined at 1.90 and 1.75 Å resolution (figure 2*a*; electronic supplementary material, figure S6A, table S1). SpeMreB3 adopts a canonical actin fold composed of four subdomains (IA, IB, IIA and IIB) [34] and consists of the same secondary structure elements as CcMreB, *Thermotoga maritima* (Tm) MreB and SciMreB5, except for the C-terminal region [3,4,35]. The N-terminal amphipathic helix was not modelled for either the Nf-SpeMreB3 or SpeMreB3-AMPPNP complexes because of poor electron density.

The Nf-SpeMreB3 crystal belongs to the space group *P*2_1_ and contains a single molecule in an asymmetric unit. Nf-SpeMreB3 forms protofilaments along the crystal *a* axis in the *P*2_1_ crystal. Thus, the protofilaments are arranged in an antiparallel manner (electronic supplementary material, figure S7A). The subunit arrangement in the protofilament resembles that of CcMreB, TmMreB and SciMreB5 protofilaments in their respective crystals (figure 2*a*) [3,4,35]. The interaction between IIA and IB' (with and without a prime indicating i and i-1 subunits, respectively), which has been observed in CcMreB, TmMreB and SciMreB5 protofilaments, is not found in the Nf-SpeMreB3 protofilament, whereas the IIA-IIB' interaction is conserved in Nf-SpeMreB3 (figure 2*b*). This IIA-IIB’ interaction is mediated by a hydrogen-bonding network and is stabilized by an electrostatic interaction between E285 and the N-terminal end of the α-helix starting from V216'.

The SpeMreB3-AMPPNP complex crystal includes two molecules (Mol-A and Mol-B) with an RMSD value estimated for C*α* atoms of 0.891 Å (figure 2*a*). These molecules are related by a pseudo twofold symmetry axis perpendicular to the crystal *a* axis, in an asymmetric unit. Each SpeMreB3 molecule in the asymmetric unit forms a protofilament with those in the neighbouring unit cells along the crystal *a* axis. Thus, the SpeMreB3-AMPPNP complex crystal also contains antiparallel pairs of the protofilaments (electronic supplementary material, figure S7B), although their arrangement differs from that of the Nf-SpeMreB3 crystal (electronic supplementary material, figure S7A). To quantitatively evaluate the domain opening, we measured the angles within the subdomains. The two molecules showed small differences in domain conformations, in which the angles composed of centroids of subdomains IIA-IA-IB (*ω*_1_) and the dihedral angle (*φ*) of SpeMreB3-AMPPNP complex Mol-A were 2.6° and 2.0° narrower than those of Mol-B, respectively (electronic supplementary material, figure S6C and D). Therefore, Mol-A had a slightly narrower nucleotide-binding cleft and a more flattened overall conformation compared to Mol-B (figure 2*d*). Nf-SpeMreB3 had larger *ω*_1_ and *φ* values than SpeMreB3-AMPPNP complex Mol-A (4.6° and 3.5°, respectively; electronic supplementary material, figure S6C and D), showing a wider nucleotide-binding cleft and a more uneven overall conformation (figure 2*d*). This conformational difference is similar to that between CcMreB monomers and protofilaments [36]. The angle composed of centroids of subdomains IIB-IIA-IA (*ω*_2_) was more constant than *ω*_1_ among our crystal structures (*ω*_2_ = 88.9° for SpeMreB3-AMPPNP complex Mol-A, 90.3° for Mol-B and 91.2° for Nf-SpeMreB3), indicating that major structural changes upon nucleotide binding contribute to swing the subdomain IB. These conformational changes lead to the interaction between subdomains IIA and IB', which are absent in the Nf-SpeMreB3 protofilament (figure 2*b*,*c*) [3,4,35]. The interaction in SpeMreB3-AMPPNP complex Mol-A is mediated through a hydrogen-bonding network and is stabilized by an electrostatic interaction between D289 and K70’ (figure 2*c*). The IIA-IIB’ interaction area of SpeMreB3-AMPPNP complex Mol-A is also wider than that of Nf-SpeMreB3 (figure 2*b*,*c*). As observed for Nf-SpeMreB3, E285 in the SpeMreB3-AMPPNP complex interacts electrostatically with the N-terminal end of the α-helix starting from V216'. The subunit interface area in the protofilament of SpeMreB3-AMPPNP is comparable to that of the other MreB protofilaments, whereas that of Nf-SpeMreB3 is much smaller (electronic supplementary material, figure S6B).

The subunit repeats along the protofilament in the crystal are in good agreement with those of the protofilament in the EM image (figures 1*c* and 2*a*). Therefore, we fitted the filament model in the crystal onto the 2D averaged EM image of the SpeMreB3 filament. The protofilament model of the SpeMreB3-AMPPNP complex fits well with the protofilament image in an antiparallel manner (figure 2*e*; electronic supplementary material, figure S6E), suggesting that the protofilament structure in the double-stranded filament (figure 1*c*) is the same as that in the crystal. However, none of the antiparallel protofilament pairs in the crystal fit onto the double-stranded filament in the EM image (electronic supplementary material, figure S7C and D), indicating that the interaction that stabilizes the double-stranded filament in solution differs from that in the crystal.

### 2.3. The ‘E ^…^ T - X - [D/E]' motif is involved in P_i_ release from SpeMreB3 and SpeMreB5

**Figure 3.**
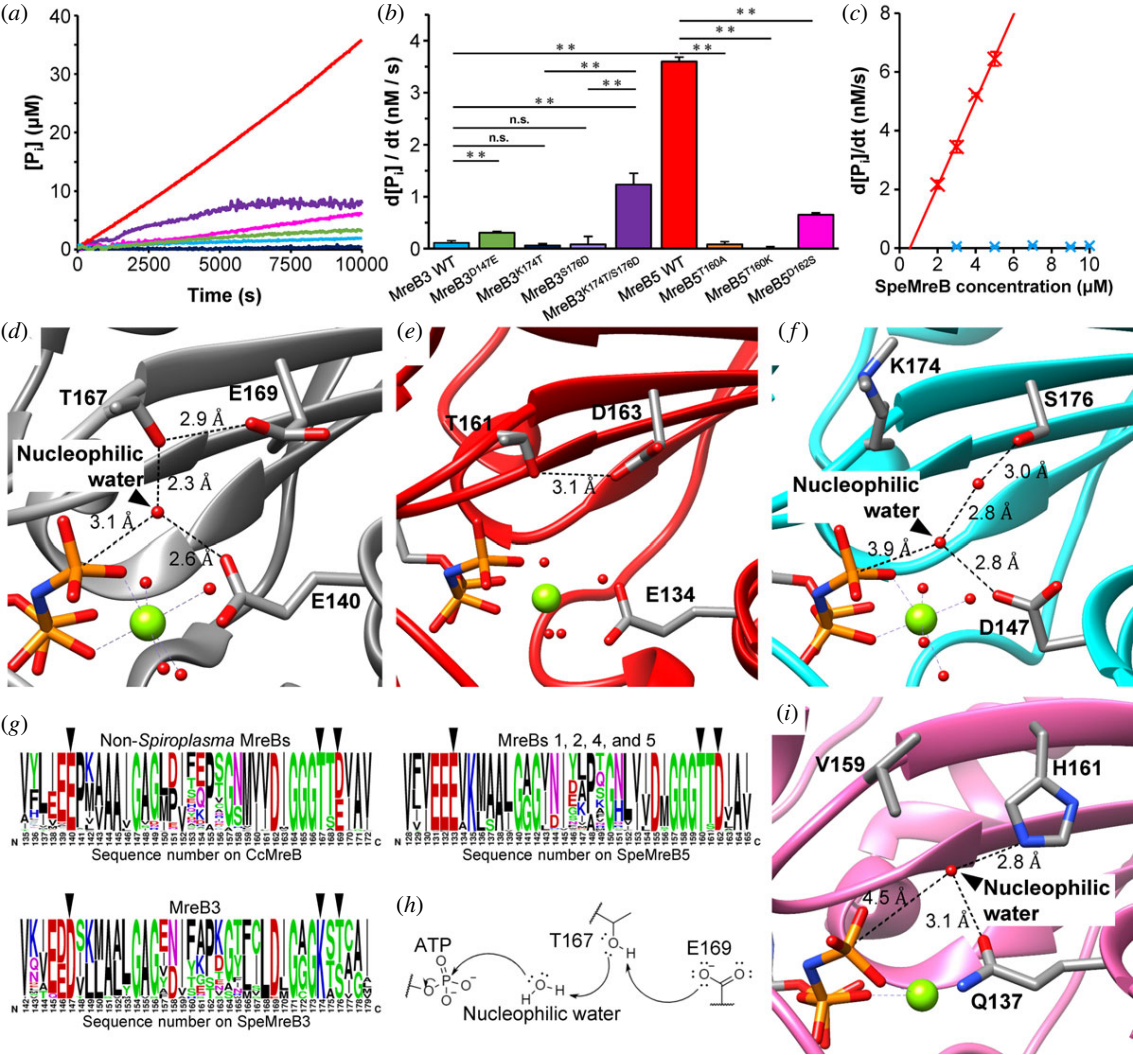
P_i_ release measurement and determination of active site structures of SpeMreB3 and SpeMreB5. (*a*) Time course plots of P_i_ release from 3 µM SpeMreB3 wild-type (cyan), SpeMreB3 D147E (light green), SpeMreB3 K174T (navy blue), SpeMreB3 K174T/S176D (purple), SpeMreB5 wild-type (red) and SpeMreB5 D162S (pink) in the presence of 2 mM ATP. The measurements were performed three times, and a representative curve is plotted in the graph. (*b*) P_i_ release rates from SpeMreBs estimated from (*a*) and electronic supplementary material, figure S8H and I. Error bars indicate the standard deviation (s.d.) from three repeated measurements. Symbols indicate *p*-value supported by Student's *t*-test (** *p* < 0.01, n.s. *p* > 0.05). (*c*) Concentration dependence of P_i_ release from SpeMreB3 (cyan) and SpeMreB5 (red). Error bars indicate the s.d. from three repeated measurements. (*d–f*) Close up view of the active sites of (*d*) the CcMreB AMPPNP complex (PDB: 4CZJ), (*e*) the SciMreB5 AMPPNP complex (PDB: 7BVY), and (*f*) the SpeMreB3 AMPPNP complex (Mol-A). Mg^2+^ and water molecules are indicated as green and red spheres, respectively. (*g*) Weblogos of amino acid sequences around the ATP hydrolysis region from: (left upper) 4832 MreB family proteins from non-*Spiroplasma* bacteria used in our previous study [9], (left lower) 29 *Spiroplasma* MreB3, and (right) 171 *Spiroplasma* MreBs (except for MreB3). The corresponding amino acid for the core amino acids motif for ATP hydrolysis (E140, T167 and E169 in CcMreB) are indicated by triangles. (*h*) A working model for ATP hydrolysis in MreB family proteins. Residues corresponding to T167 and E169 in CcMreB, the nucleophilic water molecule, and the γ-P_i_ of ATP are shown in the model. The unshared electron pairs of each atom on the residues and the water are indicated by two neighbouring dots. A putative electron transfer pathway is indicated by arrows. (*i*) Close up view of the active sites of AMPPNP-bound F-actin (PDB: 6DJM). Mg^2+^ and water molecules are indicated as green and red spheres, respectively.

Next, we measured ATPase activity of SpeMreB3 and SpeMreB5 using a P_i_ release assay. The reactions were initiated by adding a mixture of MgCl_2_, ATP and 2-amino-6-mercapto-7-methylpurine riboside (MESG), a molecular probe for P_i_ [8,37–39], to SpeMreBs in standard buffer. SpeMreB5 hydrolysed ATP and released P_i_ over time (figure 3*a,b*). The P_i_ release rate constant was 1.5 ± 0.2 nM (P_i_)/s/μM (protein), as estimated from the linear-fit slope of SpeMreB5 concentration-dependent P_i_ release rates (figure 3*c*). This value is consistent with those of SciMreB5, TmMreB, EcMreB and actin (electronic supplementary material, table S2) [5,38,40–42]. However, the P_i_ release of SpeMreB3 was too slow to estimate the rate constant, even at 10 µM (figure 3*a–c*; electronic supplementary material, table S2), a concentration at which we observed filaments using EM (figure 1*a*; electronic supplementary material, figure S3C and D).

To elucidate the structural basis of the slow P_i_ release rate, we compared the active site structures of CcMreB (PDB: 4CZJ), SciMreB5 (PDB: 7BVY) and SpeMreB3 all complexed with AMPPNP (figure 3*d–f*). In the CcMreB structure, E140 and T167 coordinate with a putative nucleophilic water molecule that attacks the γ-P_i_ of ATP. T167 also forms a hydrogen bond with E169, which is thought to be unrelated to ATP hydrolysis (figure 3*d*) [4]. These residues are structurally conserved in SciMreB5 (E134, T161 and D163 in figure 3*e*), although no nucleophilic water molecules were observed in the SciMreB5 structure. In the SpeMreB3 structure, D147 and K174 are located at positions corresponding to E140 and T167 in CcMreB, respectively. However, D147 is far from the putative nucleophilic water molecule and K174 does not interact with water molecules. The residue corresponding to E169 is replaced by serine (S176) in SpeMreB3, which does not interact with K174 (figure 3*f*). Therefore, the slow P_i_ release rate of SpeMreB3 can be attributed to these three residues.

To elucidate the role of these residues in SpeMreBs ATPase activity, we designed four SpeMreB3 variants (D147E, K174T, S176D and K174T/S176D) and three SpeMreB5 variants (T160A, T160K and D162S, which are equivalent to the T161A, T161K and D163S mutations on SciMreB5, respectively (electronic supplementary material, figure S1C)). All the variants formed filamentous structures (electronic supplementary material, figure S8A–G). The P_i_ release rate of SpeMreB3 D147E was higher than that of the wild-type. P_i_ release by SpeMreB3 K174T and SpeMreB3 S176D was similar to that of the wild-type, whereas P_i_ release by SpeMreB3 K174T/S176D was as high as 1.2 nM/s at 3 µM protein concentration (figure 3*a*,*b*; electronic supplementary material, figure S8H). The P_i_ release rates of SpeMreB5 T160A, T160K and D162S were at least 5.6-fold slower than those of the wild-type (figure 3*a*,*b*; electronic supplementary material, figure S8I). These results indicate that the amino acid motif ‘E ^…^ T - X - [D/E]' is important for P_i_ release from SpeMreBs and that the Thr–Asp/Glu pair plays a role in ATPase activity that is distinct from that the first glutamate in the motif.

To determine whether ‘E ^…^ T - X - [D/E]' is conserved in MreB family proteins, we analysed the amino acid sequences of MreBs from all bacterial phyla (figure 3*g*). The motif was conserved in 95.8% of MreB family proteins in non-*Spiroplasma* species and in 98.2% of *Spiroplasma* MreBs, except for MreB3 (figure 3*g*, left upper and right). In all known *Spiroplasma* MreB3 [9], the residues corresponding to E140 and T167 in CcMreB were replaced with aspartate and lysine, respectively. Moreover, the residue corresponding to E169 in CcMreB was replaced with serine or threonine (figure 3*g*, lower left). These findings suggest that the ‘E ^…^ T - X - [D/E]' motif is important for P_i_ release by MreB family proteins, except for *Spiroplasma* MreB3.

### 2.4. Critical concentrations of SpeMreB3 and SpeMreB5 and their variants

**Figure 4.**
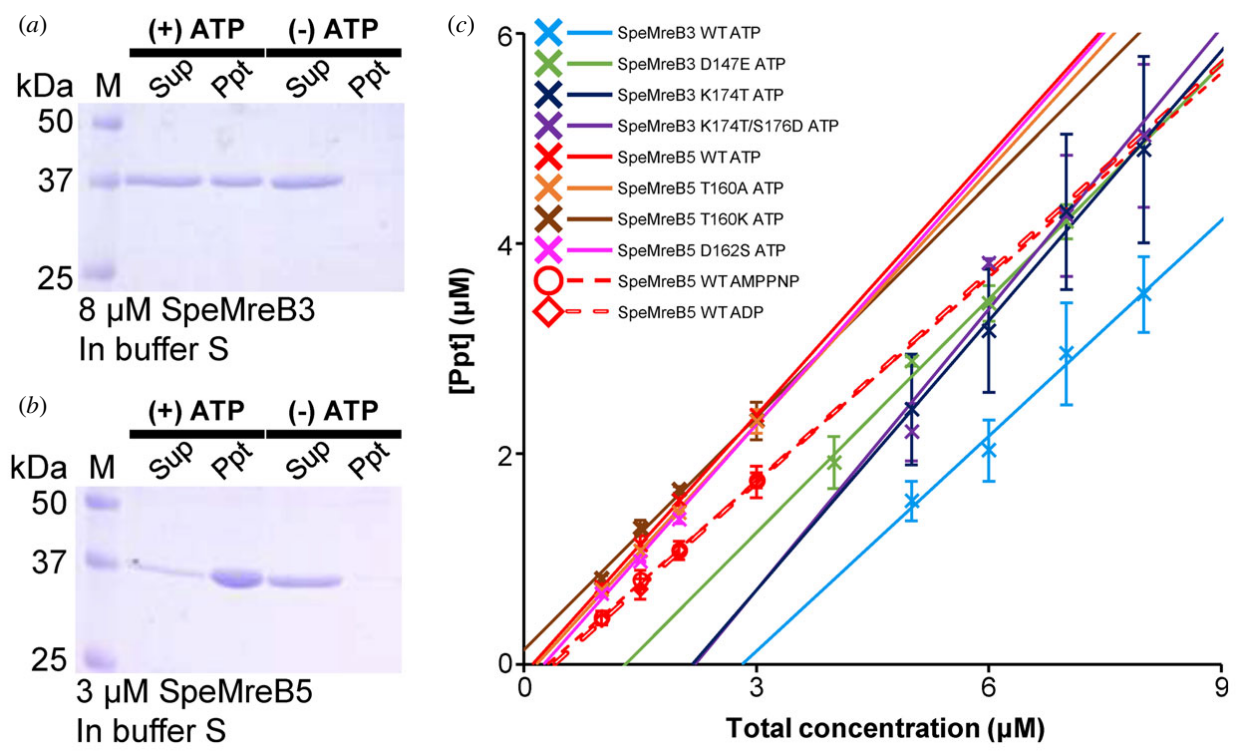
SpeMreB sedimentation assays. Each SpeMreB was incubated with buffer S (20 mM Tris–HCl pH 8.0, 1 M NaCl, 200 mM Arginine-HCl pH 8.0, 5 mM DTT, 2 mM MgCl_2_ and 2 mM ATP) for 1 h after initiating polymerization and were ultracentrifuged at 436 000×*g* for 120 min at 23°C. Precipitates were resuspended with water equivalent to the sample amount. For SpeMreB3 and its variants, each fraction was diluted three times before the preparation of the sample for SDS-PAGE. Each fraction was loaded onto a 12.5% Laemmli gel and stained with Coomassie brilliant blue to quantify the protein concentration. Fractions derived from the same sample were loaded onto adjacent lanes, and the total concentration of the sample is indicated on the lanes. (*a,b*) Sedimentation assay of (*a*) 8 µM SpeMreB3 and (*b*) 3 µM SpeMreB5 in the presence (left half lanes of each panel, (+) ATP) or absence (right half lanes of each panel, (−) ATP) of Mg-ATP. Protein size standards are visualized in Lane M, with the molecular masses of each band on the left side. (*c*) Quantified precipitation amounts of sedimented SpeMreBs. The resulting concentrations of the precipitated fractions were plotted over the total SpeMreB concentrations with linear fitting. Error bars indicate the s.d. from five repeated measurements for SpeMreB5 wild-type polymerized with ATP and ATP-analogues and three repeated measurements for the others. Critical concentrations were estimated as the *x*-intercept of each linear fit and are summarized in table 1.

To evaluate SpeMreB3 and SpeMreB5 polymerization activity, we measured their critical concentrations, which reflect the minimum concentration required for polymerization and the steady-state filament amounts, by sedimentation assays. However, significant amounts of proteins precipitated even without nucleotides in standard buffer used for EM observation and P_i_ release assay, while no filamentous structure was observed by EM in the Nf condition (electronic supplementary material, figure S3A and B, figure S9A), suggesting that the amorphous aggregation affected the measurements. Therefore, we searched for a solution in which the proteins did not form aggregates without Mg-ATP but polymerized in the presence of Mg-ATP. We found that a solution containing 20 mM Tris–HCl pH 8.0, 1 M NaCl, 200 mM L-arginine-HCl pH 8.0 and 5 mM DTT (buffer S) was suitable for sedimentation assays (figure 4*a*,*b*; electronic supplementary material, figure S3G and H).

**Table 1.** Bulk critical concentrations of SpeMreBs polymerized with buffer S as measured by sedimentation assays. The values are indicated as the mean ± s.d. from five repeated measurements for SpeMreB5 wild-type polymerized with ATP and ATP-analogues and three repeated measurements for the others except for SpeMreB3 wild-type polymerized with AMPPNP or ADP and SpeMreB3 S176D whose critical concentrations could not be determined due to the low amounts of pellets. The row below the critical concentrations indicates the *p*-value of the critical concentration compared to the associated wild-type protein polymerized with ATP, as calculated by Student's *t*-test. *p < 0.05, **p < 0.01 and n.s., *p* > 0.05.

	SpeMreB3 WT ATP	SpeMreB3 WT AMPPNP	SpeMreB3 WT ADP	SpeMreB3 D147E ATP	SpeMreB3 K174T ATP	SpeMreB3 S176D ATP	SpeMreB3 K174T/S176D ATP	SpeMreB5 WT ATP	SpeMreB5 WT AMPPNP	SpeMreB5 WT ADP	SpeMreB5 T160A ATP	SpeMreB5 T160K ATP	SpeMreB5 D162S ATP
C_C_ (μM)	2.80 ± 0.34	N.D.	N.D.	1.26 ± 0.66	2.20 ± 0.50	N.D.	2.15 ± 0.43	0.14 ± 0.07	0.29 ± 0.11	0.39 ± 0.09	0.15 ± 0.15	≈ 0	0.07 ± 0.03
*t-*test (versus WT ATP)	—	—	—	*(0.02)	n.s. (0.16)	—	n.s. (0.11)	—	*(0.03)	** (0.001)	n.s. (0.87)	—	n.s. (0.20)

To estimate the time required to a reach steady state, protein samples in buffer S were incubated with 2 mM Mg-ATP for 1, 3 and 6 h and centrifuged. No significant differences were found in pellet amounts (electronic supplementary material, figure S9B), indicating that SpeMreB polymerization reached a steady state within 1 h. Therefore, sedimentation assays were conducted after incubation for 1 h.

The critical concentration of SpeMreB5 was estimated to be 0.14 ± 0.07 µM which is comparable to that of actin polymerized in a standard buffer for the actin polymerization, KMEI buffer (0.12–0.24 µM), and of walled-bacterial MreBs in buffers akin to KMEI buffer (0.5 µM) (figure 4*c*; electronic supplementary material, figure S9D, table 1) [7,8,43,44]. By contrast, the critical concentration of SpeMreB3 was estimated to be 18 times higher than that of SpeMreB5 (figure 4*c*; electronic supplementary material, figure S9C, table 1). In actin sedimentation assays, the actin concentration in the supernatant fraction is consistent with the critical concentration [45]. However, for SpeMreB3 and SpeMreB5, the concentrations in the supernatant fractions were not constant and the linear-fit slope of the pellet amounts was not 1 (figure 4*c*).

Next, we determined the critical concentrations of SpeMreB3 and SpeMreB5 variants used in the P_i_ release assay. SpeMreB3 D147E showed a 2.2-fold lower critical concentration than wild-type SpeMreB3. SpeMreB3 S176D polymerized less than the wild-type, so we could not determine the critical concentration because of the low pellet amounts. The critical concentration of SpeMreB5 T160K was estimated to be approximately 0 µM, because it formed higher amounts of precipitates than the wild-type. These results suggest that these mutations affect SpeMreB3 and SpeMreB5 polymerization activity. By contrast, the critical concentrations of the other variants (SpeMreB3 K174T, SpeMreB3 K174T/S176D, SpeMreB5 T160A and SpeMreB5 D162S) were not significantly different from those of their respective wild-types (figure 4*c*; electronic supplementary material, figure S9C–D; table 1), suggesting that the filament amounts of these variants were not significantly different from those of the corresponding wild-types.

### 2.5. ATP hydrolysis enhances SpeMreB polymerization activity

To analyse the relationship between ATPase activity and polymerization dynamics, we determined the critical concentrations of SpeMreB3 and SpeMreB5 in the presence of Mg-ADP or Mg-AMPPNP. SpeMreB3 polymerized with AMPPNP or ADP pelleted less than those polymerized with ATP. It was difficult to determine the critical concentrations because the pellet amount was too low to be applied to linear fitting (electronic supplementary material, figure S9E). The critical concentration of SpeMreB5 was lowest in the presence of ATP and was two- and three-fold higher in the presence of AMPPNP and ADP, respectively (figure 4*c*; electronic supplementary material, figure S9F; table 1). These results suggest that SpeMreB3 and SpeMreB5 in the pre-hydrolysis and post-P_i_ release states are unstable as filaments.

## 3. Discussion

### 3.1. A model for SpeMreB polymerization dynamics

**Figure 5.**
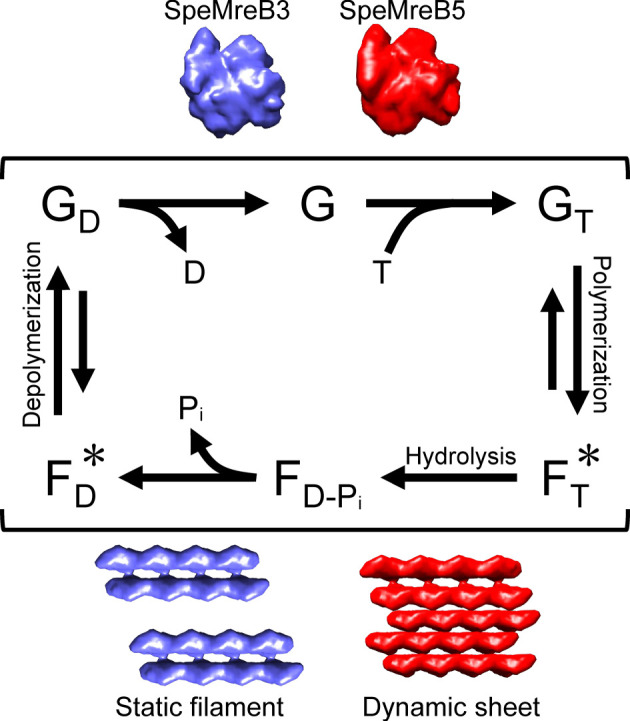
Working model of SpeMreB polymerization. ATP and ADP are denoted as ‘T' and ‘D', respectively. The characters ‘G' and ‘F' indicate SpeMreBs in the monomeric and polymerized states, respectively, named analogous to actin states. Bound nucleotides on SpeMreBs are indicated as subscripts. In the filamentous states, the pre-hydrolysis and post-P_i_ release states, which showed high critical concentrations are indicated with asterisks. The schematic structures of monomeric and polymerized SpeMreB3 (cyan) and SpeMreB5 (red) are indicated beside the corresponding positions on the polymerization cycles with the filament characters.

EM and sedimentation assays of SpeMreB3 and SpeMreB5 revealed the necessity of nucleotide binding for polymerization and unstable nucleotide states as filaments (figures 1 and 4; electronic supplementary material, figure S3). Based on these results, we construct a working model for SpeMreB3 and SpeMreB5 polymerization dynamics (figure 5). First, SpeMreBs bind ATP and polymerize into filaments, while the pre-hydrolysis state is unfavourable for them to remain as filaments (figure 4*c*; electronic supplementary material, figure S9E,F; table 1). Polymerized SpeMreBs hydrolyse ATP. Then, SpeMreBs in the ADP-P_i_ state release phosphate after a certain duration and are converted to a state with an increased critical concentration, the ADP-bound state, possibly leading to depolymerization. Eventually, the depolymerized SpeMreBs in the ADP state replace ADP with ATP and return to the initial state in the polymerization cycle.

This model is qualitatively applicable to SpeMreB3 and SpeMreB5, although their rate constants differ from each other. While our Nf-SpeMreB3 crystal structure forms a protofilament, we suppose that ATP binding is necessary to polymerize SpeMreB3, since filaments were not observed in the absence of nucleotides under solution conditions (figures 2*a* and 4*a*; electronic supplementary material, figure S3A and B). Although some intra-protofilament interactions along the filament axis were observed in Nf-SpeMreB3, the interaction area is smaller than that in SpeMreB3-AMPPNP (figure 2*b*,*c*; electronic supplementary material, figure S6B). This difference is derived from domain closure upon AMPPNP binding (figure 2*d*), suggesting that this conformational change is necessary to form filaments in solution. In contrast to our model, the ATP hydrolysis-deficient variants used in this study except for SpeMreB3 S176D showed lower or similar critical concentrations compared with the corresponding wild-types (figure 4*c*; table 1). This result may be caused by subtle differences in active site structures between the variants and the corresponding wild-types, which can change the overall conformation, thereby leading to different polymerization ability [4,40].

The working model is qualitatively consistent with that of other actin superfamily proteins [30]. However, the equilibrium balance of polymerization dynamics is probably uncommon. The critical concentration of actin polymerized with ADP is 18-fold higher than that of actin polymerized with ATP [44,46]. By contrast, the critical concentrations of SpeMreBs polymerized with ADP were only approximately two times higher than those polymerized with ATP (figure 4*c*; electronic supplementary material, figure S9E,F; table 1), suggesting that SpeMreB3 and SpeMreB5 depolymerization is not significantly stimulated by P_i_ release compared to actin filaments. Structural differences between actin and SpeMreB may cause differences in their critical concentrations. In the actin filament, the D-loop in subdomain IB (subdomain 2 in actin nomenclature) forms a major intra-protofilament interaction that is attenuated upon P_i_ release [47]. SpeMreB3 and SpeMreB5 lack a loop corresponding to the D-loop of actin, as well as MreB of walled bacteria, and their intra-protofilament interaction via subdomain IB is less than that of actin (figure 2*c*) [3,4,35]. These findings suggest that SpeMreB subunit turnover rates are slower than those of actin. This may be a fundamental feature of MreB family proteins, because the critical concentration of TmMreB polymerized with ADP is approximately twofold higher than that polymerized with ATP, as well as SpeMreB5 [8].

### 3.2. ATP hydrolysis mechanism of MreB family proteins

Our P_i_ release assays for SpeMreB3 and SpeMreB5 identified two players in ATPase activity—the threonine–acidic residue pair and the conserved glutamate corresponding to E140 in CcMreB (figure 3; electronic supplementary material, figure S8H,I). These residues form a hydrogen-bonding network with a putative nucleophilic water molecule in the CcMreB structure (figure 3*d*), suggesting that these residues play a role in ATP hydrolysis. E140 and T167 in CcMreB align the nucleophilic water into an appropriate position for ATP hydrolysis [4]. Previously, it was not clear which residue plays a role in proton elimination from the water molecule, which is a necessary step for ATP hydrolysis. Based on our findings, we propose a hypothesis for the role of active-site residues in ATP hydrolysis using CcMreB as a model (figure 3*h*). E169 eliminates the proton of the T167 side chain hydroxy group to activate this residue as an acidic catalyst for proton elimination from the nucleophilic water. E140 adjusts the position of the nucleophilic water to be suitable to attack the γ-P_i_ of ATP (figure 3*d*). This reaction mechanism is similar to that proposed for skeletal actin, where Q137 (corresponding to E140 in CcMreB) is responsible for positioning the nucleophilic water, while H161 (corresponding to E169 in CcMreB) is responsible for the proton elimination from the nucleophilic water (figure 3*i*) [47]. The amino acid motif ‘E ^…^ T - X - [DE]' is mostly conserved in MreB family proteins, except for *Spiroplasma* MreB3 (figure 3*g*), suggesting that the ATP hydrolysis mechanism proposed here is conserved in most MreB family proteins.

### 3.3. Proposed roles of SpeMreB3 and SpeMreB5 on *Spiroplasma* swimming

A previous study of *S. poulsonii* MreBs using a heterologous expression system suggested that MreB3 and MreB5 play distinct roles in the cell [26]. Our results show that SpeMreB3 and SpeMreB5 polymerize into different filamentous structures with distinct ATPase activities and critical concentrations (figures 1, 3 and 4). These differences in polymerization characteristics may be related to the differences in the roles of SpeMreB3 and SpeMreB5. MreB5 is likely an actuator that changes the conformation of the internal ribbon structure to drive the cell [26]. Our EM observations revealed that SpeMreB5 forms sheet structures with two patterns of inter-protofilament interactions (figure 1*e*; electronic supplementary material, figure S4J). These interactions were not observed in the walled-bacterial MreBs. Because of these distinct interaction patterns, the sheet exhibit both parallel and antiparallel alignment of the two protofilaments. Parallel alignment can lead to anisotropy in the overall sheet structure which meets the requirement of directional movement of *Spiroplasma* swimming. Interestingly, we did not find a sheet structure with an interior antiparallel filament, although both sides of the filament should be structurally identical. This suggests that SpeMreB5 has a mechanism that leads to the structural anisotropy of the sheet. One possible explanation is that the growth of parallel protofilaments may change the conformation of the free side of the antiparallel filament to reduce the affinity of the parallel protofilament. Another possibility is that the binding rate of a new protofilament may be much faster on the parallel protofilament than on the free side of the antiparallel pair. MreB3 likely anchors MreB1 and/or MreB4 filaments to the membrane to form a fixed structure [26]. This role does not require filament polarity. SpeMreB3 forms a double-stranded filament with possible antiparallel polarity (figure 1*c*), which is consistent with this requirement.

SpeMreB3 exhibits low ATPase activity (figure 3*a*,*b*; electronic supplementary material, figure S8H). To the best of our knowledge, this is one of the lowest activities in the actin superfamily proteins (electronic supplementary material, table S2) [5,38,40–42,48–50]. The amino acid residues responsible for low ATPase activity are conserved in MreB3 (figure 3*g*, centre). These findings suggest that low ATPase activity is involved in SpeMreB3 function. Since subunit turnover requires the completion of the ATPase cycle (figure 5), low ATPase activity leads to slow subunit turnover, suggesting that the SpeMreB3 filament is stable and may stably anchor the SpeMreB1 and/or SpeMreB4 filaments onto the membrane. This is consistent with a previous study that showed that *S. poulsonii* MreB3 forms static filaments in a heterologous expression system [26].

### 3.4. Effect of SpeMreB3 methylation on polymerization

Lysine residues were methylated to obtain SpeMreB3 crystals suitable for structural determination (electronic supplementary material, figure S5A and B). Similar to unmethylated filaments, methylated SpeMreB3 polymerized into double-stranded filaments (electronic supplementary material, figure S5C). The P_i_ release rate of methylated SpeMreB3 was also low (electronic supplementary material, figure S5D). However, in sedimentation assays using 8 µM methylated SpeMreB3, pellets were not detected (electronic supplementary material, figure S5E), indicating that polymerization activity is attenuated by methylation. One reason may be that methylation inhibits the lysine residue-mediated interactions involved in filament formation. Our crystal structure of the SpeMreB3 AMPPNP complex showed that K70, which is involved in intra-protofilament interactions, is di-methylated (figure 2*c*). In methylated SpeMreB3, this interaction may be disturbed in solution, most likely because of a decrease in the degree of freedom of the interaction. In the SpeMreB3 AMPPNP complex, K174 is di-methylated. We cannot rule out the possibility that this methylation changes the position of the residue, thus preventing interaction with the nucleophilic water (figure 3*f*) and slowing the ATP hydrolysis rate, as suggested by the decreased pellet amount in the sedimentation assay (figure 3*f*; electronic supplementary material, figure S5E). However, this methylation is unlikely to affect the above discussion on ATP hydrolysis because the P_i_ release rate was not changed by introducing the single K174T mutation (figure 3*a*,*b*,*f*; electronic supplementary material, figure S8B).

## 4. Conclusion

In this study, we clarified the distinct features of SpeMreB3 and SpeMreB5, which likely play different roles in *Spiroplasma* swimming [26]. SpeMreB3 polymerizes into a double-stranded filament, whereas SpeMreB5 forms asymmetric sheet structures (figure 1). The ATPase and polymerization activities of SpeMreB5 were higher than those of SpeMreB3 (figures 3 and 4). The low ATPase activity of SpeMreB3 was caused by the lack of the core amino acid motif ‘E ^…^ T - X - [DE],' which is conserved in the catalytic centre of most MreB family proteins (figure 3). These results indicate that *Spiroplasma* has diversified MreB characteristics to acquire unique swimming motility. Our results suggest two features presumably common to MreB family proteins: the ATP hydrolysis mechanism including a proton elimination step from the nucleophilic water molecule (figure 3), and the coupling of ATPase activity and polymerization dynamics (figure 5). These findings will shed light on the chemistry and relationship between polymerization and the cellular functions of MreB family proteins.

## 5. Material and methods

### 5.1. SpeMreB cloning and expression

The DNA sequences of SpeMreB1 to SpeMreB5 [23] were codon-optimized for *E. coli* expression and were individually synthesized by fusion with pUC57 (GenScript, Piscataway, NJ, USA). The DNA fragments encoding the SpeMreBs were excised using NdeI and BamHI restriction enzymes and inserted into pET-15b (Novagen, Madison, WI, USA) or pCold-15b, which was constructed from pCold I (Takara Bio Inc., Kusatsu, Japan) by replacing the histidine tag and proteinase digestion site sequences with that of pET-15b. Each construct was transformed into *E. coli* BL21 (DE3) and C43 (DE3) cells. *E. coli* carrying the constructed plasmid were grown overnight in LB medium in the presence of 50 µg ml^−1^ ampicillin at 37°C. The culture was then diluted with fresh medium and incubated at 37°C. When the OD_600_ value reached 0.4–0.6, IPTG was added to a final concentration of 1 mM and the cultures were incubated for 24 h at 15°C. Cells were harvested, washed twice with PBS (10 mM Na_2_HPO_4_, 2 mM NaH_2_PO_4_, 3 mM KCl and 137 mM NaCl), and stored at −80°C until further use.

### 5.2. SpeMreB purification

The wild-type and its variants of SpeMreB3 were purified by fusion with a 6 × histidine tag at the N-terminus as follows. Cell pellets harvested from 1-L cultures were resuspended in 20–40 mL buffer A (50 mM Tris–HCl pH 8.0 at 25°C, 300 mM NaCl and 50 mM imidazole-HCl pH 8.0 at 25°C) and sonicated with a probe sonicator (Nissei, Ultrasonic Homogenizer). The cell lysate was then centrifuged (100 000×*g* at 4°C for 30 min). The supernatant was loaded onto a HisTrap HP column (Cytiva, Wauwatosa, WI, USA), washed with 10 column volumes of buffer A, and eluted with 13 mL of arranged buffer A containing 230 mM imidazole-HCl pH 8.0 at 25°C. The eluted SpeMreBs were further purified using a HiLoad 26/600 Superdex 200 pg column (Cytiva) at 4°C equilibrated with buffer B (20 mM Tris–HCl pH 8.0 at 25°C and 300 mM NaCl). For SpeMreB5 and its variants, the centrifugation strength was decreased to 12 000×*g*. For purification of SpeMreB3 for the crystallization experiments, buffer C (10 mM Tris–HCl pH 8.0 at 25°C and 150 mM NaCl) was used for gel filtration with a HiLoad 26/600 Superdex 200 pg column instead of buffer B. Protein concentrations were determined from the absorbance at 280 nm measured using NanoDrop One (Thermo Fisher Scientific) with the following absorption coefficients: 0.474 (mg mL^−1^)^−1^ cm^−1^ for SpeMreB3 and its variants and 0.578 (mg mL^−1^)^−1^ cm^−1^ for SpeMreB5 and its variants.

### 5.3. SpeMreB3 methylation

SpeMreB3 eluted from Ni^2+^-NTA chromatography was subjected to HiPrep 26/10 Desalting column (Cytiva) equilibrated with buffer D (50 mM HEPES-NaOH pH 7.5 at 25°C and 250 mM NaCl) or dialysed with buffer D to replace the buffer. To methylate lysine residues in SpeMreB3, dimethylamine-borane complex (DMAB) (Merck) and formaldehyde (Merck) were added at final concentrations of 20 and 40 mM, respectively [32,33], and the sample was incubated for 2 h at 4°C. The methylated sample was desalted with a HiPrep 26/10 Desalting column equilibrated with buffer D to remove excess DMAB and formaldehyde, concentrated to less than 13 mL using an Amicon Ultra 10 K dialysis cassette (Merck), and subjected to gel filtration chromatography on a HiLoad 26/600 Superdex 200 pg column. The column was equilibrated with buffer B at 4°C. The methylation ratio of methylated SpeMreB3 reached 96.5 ± 2.2%, as confirmed by MALDI-TOF mass spectrometry (electronic supplementary material, figure S5B). To prepare methylated SpeMreB3 for crystallization, the following three steps were modified from the method described above: (1) after Ni^2+^-NTA affinity chromatography, the mixture of the sample and 100 units of thrombin (Cytiva) was dialysed overnight at 4°C in buffer D to cleave the histidine tag from SpeMreB3. (2) after 2 h of incubation with 20 mM DMAB and 40 mM formaldehyde, two incubation steps were added before the removal of excess DMAB and formaldehyde. DMAB and formaldehyde were added at concentrations of 40 mM and 80 mM, respectively, and the sample was incubated for 2 h at 4°C. Then, an additional 10 mM DMAB (total 50 mM) was added and the sample was incubated overnight at 4°C. In this procedure, although the terminal 7–13 amino acids of methylated SpeMreB3 were cleaved, and small amounts of degraded products appeared even after the final purification step, methylated SpeMreB3 was successfully crystallized. (3) Buffer C was used for gel filtration on a HiLoad 26/600 Superdex 200 pg column instead of buffer B.

### 5.4. SpeMreB polymerization

To prepare samples for EM observations and to measure P_i_ release rates, SpeMreBs were polymerized in standard buffer (20 mM Tris–HCl pH 7.5 at 25°C, 100 mM KCl, 5 mM DTT, 2 mM MgCl_2_ and 2 mM ATP). For sedimentation assays, SpeMreBs were polymerized in buffer S (20 mM Tris–HCl pH 8.0 at 25°C, 1 M NaCl, 200 mM L-arginine-HCl pH 8.0, 5 mM DTT, 2 mM MgCl_2_ and 2 mM ATP). Prior to polymerization, the SpeMreB buffer was replaced from buffer B to the desired buffer in the absence of DTT, MgCl_2_ and ATP by overnight dialysis at 4°C with a buffer volume 50–100 times higher than that of the sample solution. Monomeric SpeMreBs with a concentration lower than the desired concentration were concentrated using Amicon Ultra 10 K cassettes. The samples were centrifuged to remove aggregates. Then, DTT, MgCl_2_ and ATP were added to initiate polymerization. All polymerization reactions were performed at room temperature (approximately 25°C).

### 5.5. Electron microscopy

The SpeMreBs were polymerized for 3 h, which was long enough to obtain steady state samples for the other MreBs [5–8,41,51]. A sample (4 µL) was placed onto a 400-mesh copper grid coated with carbon for 1 min at room temperature, washed with 10 µL of water, stained for 45 s with 2% (w/v) uranyl acetate, air-dried, and observed under a JEOL JEM-1010 transmission electron microscope (Tokyo, Japan) at 80 kV equipped with a FastScan-F214T charge-coupled device camera (TVIPS, Gauting, Germany). To obtain SpeMreB3 and SpeMreB5 images for 2D averaging, 10 µM SpeMreB3 and 5 µM SpeMreB5 polymerized with standard buffer were diluted to 3 µM immediately before sample placement onto a grid. For image averaging, SpeMreB images were automatically selected as helical objects and were segmented in a box of 128 × 128 pixels with 90% overlap using RELION v. 3.1 [31]. The images were processed using the estimation of the contrast transfer function and reference-free 2D class averaging using RELION v. 3.1 or 4.0 [31]. For the image averaging of SpeMreB3, 13 077 particles were extracted from 51 field images and classified into 50 classes, yielding a class composed of 2874 particles as the final particle set. For SpeMreB5, 117 740 particles were extracted from 70 field images and classified into 200 classes, yielding initial classes of images with two, three, four and five protofilaments. The particle sets in each class (2104, 4155, 5201 and 3992 particles for the classes of images with two, three, four and five protofilaments, respectively) were individually subjected to 2D classification into 50 classes, excluding some particles from the initial particle sets that interfered with image averaging. This step was repeated two more times with class numbers of 25 and 3 at each classification step. Three sets of SpeMreB5 images with two, three, four and five protofilaments were obtained by averaging 1593, 2211, 1191 and 928 particles, respectively (figure 1*d*,*e*; electronic supplementary material, figure S4J). The subunit repeats in the 2D averaged images were estimated as the distances between the minimal values of the greyscale profiles quantified using ImageJ (National Institutes of Health; http://rsb.info.nih.gov/ij/). To determine each subunit axis of the 2D averaged images, the images were subjected to black and white inversion and binarized with a grey value threshold of 75 (for the SpeMreB3 and SpeMreB5 filaments) and 68 (for the SpeMreB5 five-stranded sheet), in which the densities for intra- and inter-protofilament interaction regions were excluded using ImageJ. Each separated subunit density was subjected to elliptical fitting using ImageJ and the major axis was defined as the subunit axis.

### 5.6. Crystallization and structural determination

Crystallization screening was performed using the sitting-drop vapour-diffusion technique with the following screening kits: Wizard Classic I-II (Rigaku Reagents, Inc., Bainbidge Island, USA), Wizard Cryo I-II (Rigaku Reagents, Inc.), PEG/Ion Screen I-II (Hampton Research, Alison Viejo, USA), Crystal Screen I-II (Hampton Research), SaltRx I-II (Hampton Research) and PEG/Ion 400 (Hampton Research), at 4°C and 20°C. Nf-SpeMreB3 crystals used for X-ray data collection were grown at 4°C from drops prepared by mixing 0.5 µL of protein solution (5 mg mL^−1^) in buffer C with an equivalent volume of reservoir solution containing 100 mM MES-NaOH pH 6.0, 20% (w/v) PEG-8000 and 200 mM calcium acetate. The crystals belonged to the space group *P*2_1_ with unit cell dimensions of *a* = 52.4, *b* = 68.1, *c* = 54.6 Å and *β* = 91.7°. The SpeMreB3 AMPPNP complex crystals used for X-ray data collection were obtained at 20°C from drops prepared by mixing 0.5 µL of protein solution (5 mg mL^−1^) in buffer C containing 5 mM MgCl_2_ and 5 mM Li_4_AMPPNP with an equivalent volume of reservoir solution containing 100 mM acetate-NaOH pH 4.6, 30% (w/v) PEG-4000 and 200 mM ammonium acetate. The crystals belonged to the space group *P*2_1_ with unit cell dimensions of *a* = 50.3, *b* = 56.3, *c* = 120.5 Å and *β* = 90.6°.

X-ray diffraction data were measured at 100 K at synchrotron beamlines BL41XU and BL45XU at SPring-8 (Harima, Japan) with the approval of the Japan Synchrotron Radiation Research Institute (JASRI) (proposal nos. 2018A2567, 2018B2567, 2019A2550 and 2019B2550). The crystals were cryoprotected by soaking in a 1 : 9 mixture of glycerol and reservoir. The diffraction data were processed using MOSFLM [52] and scaled using Aimless software [53]. The initial phase was determined by molecular replacement (MR) with Phaser software [54] using a previously reported CcMreB structure (PDB ID: 4CZL). An atomic model of Nf-SpeMreB3 (P17-L344) was constructed using Coot [55] and refined using Phenix [54]. The refined Nf-SpeMreB3 structure was used for MR of the SpeMreB3 AMPPNP complex. An atomic model of the SpeMreB3 AMPPNP complex (Mol-A: P17–N348; Mol-B: P18–E347) was built using Coot [55] and was refined using Phenix [54]. The data collection and refinement statistics are summarized in electronic supplementary material, table S1.

### 5.7. Analyses of domain angles

The SpeMreB3 structures were divided into four subdomains according to the definition of MreBs in walled-bacteria (electronic supplementary material, figure S1B). The first 17 amino acids (M1-P17) and the last eight amino acids (I345-K352) were excluded from subdomain IA because the corresponding regions were not modelled in SpeMreB3-AMPPNP Mol-B and Nf-SpeMreB3, respectively. The centroid of each subdomain was calculated from the backbone atoms without mass-weighting on each atom using UCSF Chimera v. 1.13.1. The centroids were used for calculations of the domain opening and dihedral angles using UCSF Chimera v. 1.13.1 (electronic supplementary material, figure S6C and D).

### 5.8. P_i_ release assays

P_i_ release was measured using EnzChek kits (Thermo Fisher Scientific), in which the P_i_ concentration was traced by the absorbance of the reaction product between P_i_ and 2-amino-6-mercapto-7-methylpurine riboside (MESG), a molecular probe for P_i_, at 360 nm (A_360_) [8,37–39]. Reactions were initiated by adding a mixture of MgCl_2_, ATP and MESG to SpeMreBs in standard buffer without Mg-ATP. The A_360_ values of SpeMreBs without MESG and control buffer containing MESG were subtracted from those measured for SpeMreBs with MESG. Data were collected using a Varioskan Flash spectrophotometer (Thermo Scientific), which had an initial measurement delay of approximately 1 min.

### 5.9. Sedimentation assays

SpeMreBs with a volume of 200 µL were polymerized for 1–6 h and were centrifuged (100 000 r.p.m. at 23°C for 120 min) in a TLA-100 rotor (Beckman Coulter). The pellet was resuspended in 200 µL water. The supernatant and pellet fractions were subjected to electrophoresis on a 12.5% Laemmli gel and stained with Coomassie Brilliant Blue R-250 to determine the concentration of each fraction. The SpeMreB band intensities were quantified using ImageJ. The concentrations of the supernatant and pellet fractions were estimated as the products of the total SpeMreB concentration and the ratio of each fraction to the sum of the supernatant and pellet fractions. The critical concentration was determined as the *x*-intercept of a linear fit with the precipitate amounts at the steady state over the total SpeMreB concentration.

## Data Availability

The X-ray crystal structure and structure factors of SpeMreB3 have been deposited in Protein Data Bank under the accession code of 7E1C (Nf-SpeMreB3) and 7E1G (SpeMreB3-AMPPNP complex). The other raw data are available from the corresponding authors on reasonable request. Supplementary material is available online [56].
